# Supplementary material for: Family and personal factors predisposing adolescents to substance abuse in high-risk urban areas
Source: PLoS One. 2025 Nov 5;20(11):e0334072. doi: 10.1371/journal.pone.0334072 (PMC12588477; doi:10.1371/journal.pone.0334072)
Supplement: Attachment — Submitted filename: Response to reviewers.odt [file pone.0334072.s002.odt]

Response to reviewers


Reviewer #1
The phrase ‘substance abuse’ is utilised throughout the article, however this phrase is associated with stigma and negatively reinforces substance use and associated discrimination. It would be better to utilise ‘substance use’ throughout the article, even in the case of illicit drug use. Line 167 also states ‘substance users’, which again attaches stigma and moves away from person-centred language. Rephrasing to ‘adolescents who use drugs’ will help to eliminate potential stigma. The following NIH resource outlines appropriate terminology to reduce stigma in research: https://nida.nih.gov/research-topics/addiction-science/words-matter-preferred-language-talking-about-addiction
Substance use in this population is quite common, so we wanted to highlight that the studied population not only used but had issues related to their use. We have modified the wording to avoid stigmatisation and we now use the expresion substance use.

In terms of consent, was consent obtained from the children themselves? The emphasis is on the signature of the parents/legal guardians, but did the children also provide consent, particularly as the age range was 12-18 years old. If only parental/guardian consent was obtained, did the children feel compelled to participate, even if they did not wish to? This needs to be made clearer within the methods as the focus seems to be on parental/guardian consent.
Thanks for noting this. All participation was voluntary. We have updated the methods section to make it clear:
Only those participants who were willing to participate and returned the consents signed by their legal guardians were included in the study.

For the statistical tests, was software utilised? If so, it should be detailed within the methods section.
All analyses were conducted using R. We have updated the Analysis Plan sub-section to include this.

The results do not mention the gender of participants. Unsure if this has been omitted for a specific reason, such as protecting participants anonymity? This needs to be clarified either in the results section or within the limitations.
Thanks for noting this! We forgot to mention that all participants were male. We have updated the Participants sub-section from the methods section to clarify this. The fact that all substance users were male was unintentional. Participants from the comparative groups were chosen to match those from the substance use group.

Lines 357-359: this sentence links the study results to ‘genetic and environmental factors’ which lead to substance use. However, the study does not explore any genetic factors, merely the relationship between familial substance use and adolescent substance use. I would redraft this sentence to reflect the fact that the study does not explore genetic implications.
We agree. Thus, the sentence was rephrased to focus on the investigated variables.

The introduction and discussion alike rely on some dated studies, much of which is over 10 years old. Both sections would benefit from the inclusion of some more recent research.
Thank you for this observation. We agree that this represents a limitation of our manuscript and have therefore revised the introduction, incorporating nine recent studies published within the last decade

Reviewer #2
Line 83 – 92 - A more detailed definition of both emotional stability and cognitive control (executive function) and the interaction between these two systems would be useful in the introduction, For example the author states that emotional regulation plays a role in controlling behaviours, while cognitive function is defined as controlling actions – it is not clear how you are differentiating between behaviours and actions. Using widely accepted definitions around these elements and their interaction would solve this.
Thank you for noting this. We have revised the paragraph to clarify the relationship, explaining how both constructs interact through the use of emotion regulation strategies. We have also added relevant references on this subject. The revised paragraph now reads:

Emotional stability refers to an individual's ability to maintain a balance in their feelings and behaviours, which is crucial during adolescence—a period marked by emotional volatility and developmental changes. This is achieved through the use of emotion regulation strategies leading to wellbeing and adaptive behaviour. The engagement of adaptive emotion regulation strategies leading to emotional stability is a complex process that also involves the use of cognitive resources, particularly those involved in cognitive control [36,37,38]. Cognitive control involves the ability to regulate and manage one's thoughts and actions, which is critical in resisting impulsive behaviours such as drug use [9,10]. 

Similarly in lines 93 to 97 you state the interaction between emotional stability and cognitive control can influence substance abuse, but your example only considers the impact of emotional stability and not the role of cognitive function or the interaction. Expanding on this slightly would greatly improve the paper.
We have updated the paragraph to better explain the results from the cited study and included more evidence on the role of both processes in the development of substance use.

Line 123 – 131 is there any evidence around using these interventions with families where parents are using substances specifically? Some consideration of this should be included either here or in the discussion where recommendations are made.
We have expanded the paragraph considering current evidence on the effectiveness of such interventions.

Line 357 – 365 – some consideration /acknowledgement of the additional factor of availability of substances within the home meaning young people have increased opportunity to experiment would be beneficial here.
Thanks for this suggestion. We have acknowledged the increased risk of family members with substance use disorder in the development of related issues in adolescents and provided a relevant reference.

Line 368 – 379 – disparities in educational background should potentially be mentioned here. Particularly as there are strong known interactions between education and emotional and cognitive control.
We appreciate this observation; unfortunately, educational background data were not available for all participants, which prevented meaningful group comparisons. We added informationin the paragraph to acknowledge this limitation.
